# Supplementary figures and images for: Sex differences in sleep and influence of the menstrual cycle on women’s sleep in junior endurance athletes
Source: PLoS One. 2021 Jun 17;16(6):e0253376. doi: 10.1371/journal.pone.0253376 (PMC8211225; doi:10.1371/journal.pone.0253376)

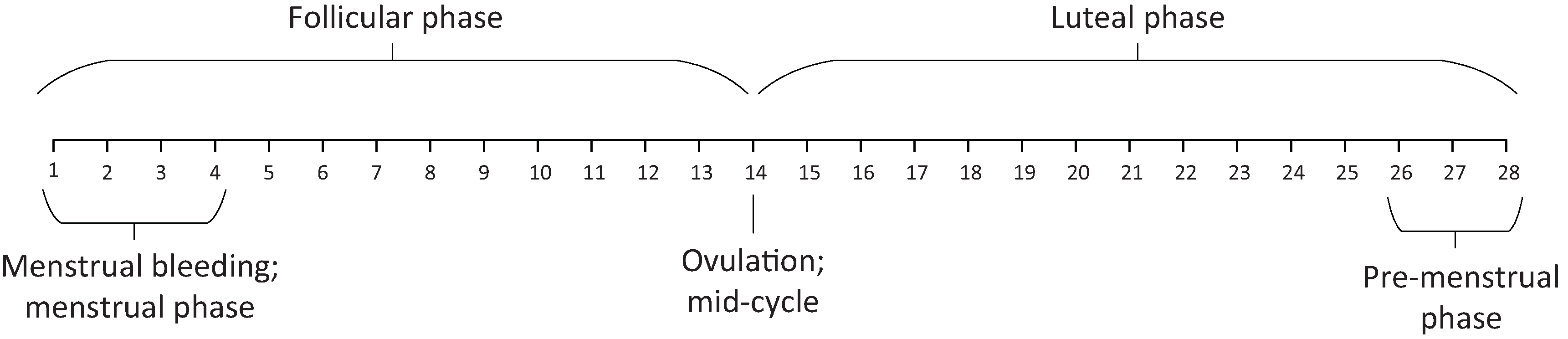

Supplement: S1 Fig — An illustrative example of the division of the menstrual cycle into menstrual, pre-menstrual, follicular and luteal phases investigated in this study. (TIF) [file pone.0253376.s001.tif]
