# Supplementary material for: Sex differences in sleep and influence of the menstrual cycle on women’s sleep in junior endurance athletes
Source: PLoS One. 2021 Jun 17;16(6):e0253376. doi: 10.1371/journal.pone.0253376 (PMC8211225; doi:10.1371/journal.pone.0253376)
Supplement: S1 Table — The intensity scale used in this study to determine the training load of endurance and strength, plyometric and speed training. (DOCX) [file pone.0253376.s002.docx]

# **Supporting Information**

| S1 Table.  *The intensity scale used to determine the training load of endurance and strength, plyometric and speed training in this study.* | | | | | | | | | | | | | |
| --- | --- | --- | --- | --- | --- | --- | --- | --- | --- | --- | --- | --- | --- |
| **5-zone Norwegian Olympic Federation’s Intensity Scale** | | | | |  | **Physiologically accurate 3-zone scale** | | |  | **TRIMP weight** |  | **Reference information** | |
| **Zone** | **Lactate (mmol/L)** | | **Heart rate (% of max.)** | |  | **Lactate turning point** | | **Intensity zone** |  |  |  | **sRPE** | **Typical training sessions** |
|  | |  | |  | | |  | | | | | | |
| **Endurance training** | | | | | | | | | | | | | |
| 1 | 0.8–1.5 | | 55–72 | |  | <1^st^ lactate threshold | | Low intensity training |  | 1 |  | 0-4 | Warm-up/cool down, >90 min |
| 2 | 1.5–2.5 | | 72–81 | |  |  |  |  |  |  |  |  | Moderate duration, 45-90 min |
| 3 | 2.5–4.0 | | 82–87 | |  | 1^st^-2^nd^ lactate threshold | | Moderate intensity training |  | 2 |  | 5-6 | Continuous sessions, 30-60 min; intervals with 6-15 min periods |
| 4 | 4.0–6.0 | | 88–92 | |  | >2^nd^ lactate threshold | | High intensity training |  | 3 |  | 7-10 | Competitions, intervals with 4-8 min periods |
| 5 | 6.0–10.0 | | 92–97 | |  |  |  |  |  |  |  |  | Competitions, intervals with 1-5 min periods |
|  | |  | |  | | |  | | | | | | |
| **Strength, plyometric and speed training** | | | | | | | | | | | | | |
| - | - | | - | |  | - | | - |  | 1.5 |  | - | Plyometric / strength exercises, 4-30 reps; speed training, 5-15 sec periods |
| *Notes*. sRPE = session rating of perceived exertion. The lactate and heart rate values refer to typical values for the training in the different intensity zones, although these parameters were not directly measured in this study. | | | | | | | | | | | | | |
