# Supplementary material for: Sex differences in sleep and influence of the menstrual cycle on women’s sleep in junior endurance athletes
Source: PLoS One. 2021 Jun 17;16(6):e0253376. doi: 10.1371/journal.pone.0253376 (PMC8211225; doi:10.1371/journal.pone.0253376)
Supplement: S2 Table — The questionnaire included 5 items, given to the participants in Norwegian, scored on a visual analogue Likert scale ranging from 0 (low well-being) to 10 (high well-being). (DOCX) [file pone.0253376.s003.docx]

| S2 Table.  *The well-being questionnaire used in the present study, with items and scale points (0 – 10) in English and Norwegian.* | | | |
| --- | --- | --- | --- |
| **Item** | | **Scale points 0 – 10** | |
| **English** | **Norwegian** | **English** | **Norwegian** |
| Energy level | Energilevel | Lacking energy – full of energy | Energiløs – energifull |
| Mood | Humør | Depressed – elated | Nedstemt – oppstemt |
| Muscle soreness | Muskelømhet | Sore muscles – “fresh” muscles | Ømme muskler – “freshe” muskler |
| Worry and rumination | Grubling og bekymring | Worried / ruminating – not worried / not ruminating | Grublende / bekymret – grublefri / bekymringsfri |
| Sleep quality | Søvnkvalitet | Poor – very good | Dårlig – meget god |
| Notes. The questionnaire was given to the participants in Norwegian, participants’ mother tongue. | | | |
