# Supplementary material for: Sex differences in sleep and influence of the menstrual cycle on women’s sleep in junior endurance athletes
Source: PLoS One. 2021 Jun 17;16(6):e0253376. doi: 10.1371/journal.pone.0253376 (PMC8211225; doi:10.1371/journal.pone.0253376)
Supplement: Attachment — Submitted filename: Response to Reviewers.docx [file pone.0253376.s005.docx]

**Response to Reviewers**

PONE-D-20-31663
Sex differences in sleep and influence of the menstrual cycle on women’s sleep in junior endurance athletes

# **Academic Editor**

| Comment | Answer |
| --- | --- |
| Please ensure that your manuscript meets PLOS ONE's style requirements, including those for file naming. The PLOS ONE style templates can be found at  <https://journals.plos.org/plosone/s/file?id=> wjVg/PLOSOne_formatting_sample_main_ body.pdf and  <https://journals.plos.org/plosone/s/file?id=b> a62/PLOSOne_formatting_sample_title_ authors_affiliations.pdf | We have now gone through the templates, and have done our best to ensure that the manuscript now adheres to the journal’s style requirements. |
| Please provide additional details regarding participant consent.  In the ethics statement in the Methods and online submission information, please ensure that you have specified what type you obtained (for instance, written or verbal, and if verbal, how it was documented and witnessed). If your study included minors, state whether you obtained consent from parents or guardians. If the need for consent for participants under 18 was waived by the ethics committee, please include this information. | We have now added additional details regarding participant consent to the manuscript. In the “Materials and Methods / Ethical Considerations” section, we now outline:   - Type of obtained consent (line 97) - Age threshold for obtaining parental consent in Norway (lines 115-118) |
| Please include additional information regarding the survey or questionnaire used in the study and ensure that you have provided sufficient details that others could replicate the analyses. For instance, if you developed a questionnaire as part of this study and it is not under a copyright more restrictive than CC-BY, please include a copy, in both the original language and English, as Supporting Information. | We have now added additional details regarding the questionnaire used in the study to the manuscript. In the “Supporting Information” section (lines 679-684), we now include the exact wording of the items of the Well-being questionnaire used in the present study, and the meaning of the scale points, both in Norwegian (the language used by the participants) as well as English. |
| In your Methods section, please provide additional information about the participant recruitment method and the demographic details of your participants. Please ensure you have provided sufficient details to replicate the analyses such as:  - the recruitment date range (month and year)  - a description of any inclusion/exclusion criteria that were applied to participant recruitment  - a table of relevant demographic details  - a description of how participants were recruited  - descriptions of where participants were recruited and where the research took place. | We have now added additional details regarding recruitment and participant information to the manuscript. In the “Materials and Methods / Participants” section, we now outline:   - the recruitment date range (line 94) - relevant exclusion criteria (lines 108-110) - demographic details in text (lines 106-107, 111-112) - a statement as to whether our sample can be considered representative of a larger population (lines 102-105) - place of recruitment and data collection (line 95) |
| Thank you for stating the following in the Acknowledgments Section of your manuscript: '..The authors acknowledge the financial support of the Centre for Elite Sports Research.' We note that you have provided funding information that is not currently declared in your Funding Statement. However, funding information should not appear in the Acknowledgments section or other areas of your manuscript. We will only publish funding information present in the Funding Statement section of the online submission form.  a. Please remove any funding-related text from the manuscript and let us know how you would like to update your Funding Statement. Currently, your Funding Statement reads as follows: 'The author received no specific funding for this work.'  b. Please include your amended statements within your cover letter; we will change the online submission form on your behalf. | We have now removed the sentence from the “Acknowledgments” section of the manuscript. The study has not received any specific funding, but was generally supported by the Centre for Elite Sports Research. Therefore, the Funding Statement as it stands now is correct. |
| We note that you have stated that you will provide repository information for your data at acceptance. Should your manuscript be accepted for publication, we will hold it until you provide the relevant accession numbers or DOIs necessary to access your data. If you wish to make changes to your Data Availability statement, please describe these changes in your cover letter and we will update your Data Availability statement to reflect the information you provide. | We now provide the data set in SPSS-format (.sav). |
| Please ensure that you refer to Figure 2 in your text as, if accepted, production will need this reference to link the reader to the figure. | The figure is now referred to in text (line 275). |

# **Reviewer 1**

| Comment | Answer |
| --- | --- |
| The authors measured sleep through radar in male and female youth athletes across 61 days. They report comparisons between male and female, and between various menstrual-cycle related time-points. Their conclusion is that females do have better objective but worse subjective sleep than males, that women spent longer time in bed and in deeper sleep during menses vs. non-menses, lower sleep efficiency during the follicular vs. luteal phase and lower light sleep in menstrual vs. pre-menstrual phase. The authors conclude recovery may be increased during menstrual and pre-menstrual times.  The manuscript is well-organised, well-written, correctly interpreted and adequately addresses limitations. The results are important, novel and applicable as well as paving the way for future research. | Thank you for the sum-up, and for a positive review of this manuscript. We have now amended the manuscript in accordance with your comments. Please consider the new version of the manuscript, where changes are shown using the track-changes function in Word. |
| Line 237, should be Figure 2 | Reviewer 2 has requested we move Figure 1 to the Supplementary materials. Therefore, the figure you are referring to is now Figure 1 (reference in text now found in line 275, and Figure 1 caption in line 297). |
| Line 307, I assume this is just supposition and not evidence-based? If the latter, please add reference. | Yes, this is a supposition and thus cannot be supported by a reference. We propose that menstrual bleeding days may have a greater impact on female athletes than non-athletic women, since female athletes have greater physical / training loads. Thus, the added load of the menstrual bleeding may have an accumulative effect in female athletes, disrupting sleep during their menstrual bleeding phase. We hope that future research can explore this possibility further. |
| The major comment, which the authors have addressed, is that they did not confirm ovulatory cycles. This does not invalidate results, but does mean they need replicating (with confirmation) and/or taking with precaution. | We fully agree with your comment, and hope that this limitation is clearly presented in the “Limitations” section of the manuscript, lines 473-500. |

# **Reviewer 2**

| Comment | Answer |
| --- | --- |
| This manuscript describes sex difference in sleep over 61 days in a small sample of young Norwegian athletes that included 15 girls who also tracked their menstrual cycles. The introduction sets up the research aims, and the purpose is clear. Methods included radar-sleep tracking (Somnofy sleep monitor developed and validated in Norway) for objective sleep duration and sleep stages. The Pittsburgh Sleep Quality Index (PSQI) was used for self-report sleep quality over the past month. Measures are well described for menstrual cycle phases by diary reports of menses days. Analyses included multivariate modeling. Results support past research that women score worse on subjective PSQI sleep quality but better on objective sleep measures. Girls had longer sleep duration than boys, and sex accounted for 25% of the variance (R-square = .25) in Total Sleep Time (TST), and 12.6% of the variance (R-square = .126) in Sleep Efficiency (SE). Sex explained 6.1% of the variance in PSQI sleep quality scores (R-square = .061). For the 15 female athletes, menstrual cycle changes in objective sleep were either less evident, with low variance explained, or sample was indeed too small to obtain statistical significance; they were in bed longer and had less sleep efficiency during the follicular phase; these two variables have obvious collinearity. Results are discussed in light of prior studies, in particular one study by Driver et al. (#21). Limitations are adequately addressed. | Thank you for the summary, and for reviewing this manuscript. You have provided us with useful feedback, which we have done our best to adhere to. Please consider the new version of the manuscript, where amendments in accordance with your comments are tracked. |
| Sleep stages defined in Table 1 are for minutes. Given the sex differences in TST, one would expect a longer sleep duration to include more minutes in each sleep stage. Standardizing each night’s time spent in sleep stages as a percentage of their TST would better support the interpretation of the results about sleep stages. | You raise a good point regarding providing the variation in sleep stages in percentages. We had this information available, and have now accordingly updated the manuscript. Data on the sleep stages provided in percentages are now provided in Table 1, line 136. We have added a “Unit” column to Table 1, to make the distinction more apparent. In addition, we have additionally run the random intercept models also using the sleep stages in percentage. Please see the updated “Results” section for the relevant statistics and visual representations in both Figure 1 and Figure 2, and the updated “Discussion” section where we discuss the new findings. |
| The Somnofy system was not particularly valid for sleep onset latency in a prior publication, but please indicate the criterion for minutes to onset – it could be lights-out to first stage of LS, first stage of SWS, or first 1, 2, 10 minutes of LS, for example. Do authors have REM onset latency as well? | SOL, as measured by the Somnofy, represents the time from lights-out to the first epoch of any sleep stage. We have now specified this further in Table 1 (line 136). We have now also added data on REM latency, to the manuscript. All analyses have been done for REM latency as well. |
| In light of the discussion, please indicate if women took pain medication during menses. | Unfortunately we did not collect data about the women’s use of pain medication. However, we did collect information about the female athletes’ menstruation-related symptoms. In this realm we asked about the symptoms they experienced during their menstrual bleeding, and whether these influenced their sleep or not. We have now added information about this to the manuscript, in the “Instruments” (lines 160-166) and “Results” sections (lines 305-308). Also we now discuss the implications of this (lines 418-423). We do, however, agree that information on pain medication would have been relevant to ask about as well. We now propose that future research should collect information on the use of pain medication to alleviate the symptoms (line 493). |
| Table 1 would be more useful to readers if it was specific to hours or minutes (rather than “time”). Because SE refers to sleep efficiency, the S.E. columns in table 2 and table 3 should be spelled out or distinguished in some way. This would be a useful place to add criterion for sleep onset latency, and to add REM onset latency. | We have now added a column “Unit” in Table 1 (line 136), which specifies the measurement unit of the different sleep variables in. REM latency has now been added to Table 1, and we also ran analyses for this variable, which are presented in the “Results” section, and subsequently discussed. For each table, we explain the abbreviation S.E. now, which we have previously left out. We hope that this clarifies the difference between SE and S.E. |
| There are 2 tables labeled as Table 2. The first table 2 is of less interest and could be supplemental. The second Table 2 with R-square values in the last column needs to be more clear – please provide the total R-square at the end, if all of these were in the same model, but it is unclear if they are separate models because of the collinearity between all these sleep variables. The R-square percentages in Table 2 or Table 3 are confusing, unless the heading in the column is meant to say "percent variance explained" rather than the actual "R-square" value. Italicizing significant p-values is very difficult to see in tables; with p-values provided, italics are not needed in that column. An asterisk or bolding would be better, depending on journal requirements. | Table 2 (showing the way we have calculated training load) has now been moved to the Supplementary materials, and is here denoted as Table S1. In the main manuscript, Table 2 is now the table showing sex differences in sleep (line 289).  Each line in Table 2 and Table 3 represents a separate model, therefore the R^2^ values are also provided separately. In other words, the first set of random intercept models regressed sex (0=man, 1=woman) on TIB, SOL, TST, LS, SWS, REM, REML and SE separately. Therefore, the R^2^ values in Table 2 show that, for instance, sex explains 27.8% of variance in REM sleep (h) specifically. The same principle was applied for the other sets of random intercept models. We have now added the sentence: “Each set of random intercept models was applied to the sleep variables separately.” (lines 263-264) in the “Statistical analyses” section of the manuscript to make this clear.  We now use bold instead of italics to highlight significant results in the tables. We explain the abbreviation R^2^ in the Notes of the tables as R^2^ = explained variance in %. |
| ICC values need a little more explanation and discussion. They are very low in Tables 2 and 3, and correlations above .80 would be expected for validity and reliability in night-to-night sleep parameter consistency. Please indicate how many nights are represented with these values and why they are important to include in results for the regression model(s). | In multilevel models, ICC values are used as an estimate of variability of the measured dependent variables within a person (be aware that in multilevel models, ICC does not represent a reliability measure). ICC above .80 indicates that there is almost no variability within a person, which is not the case for the sleep variables presented in this study. As you say, the correlations in this study are very low. This indicates that there is substantial within-level variability in the measured dependent variables. For instance, when investigating the role of sex in sleep, the ICC of SOL is .21, meaning that 21% of the variance in SOL is due to differences between participants, while 79% of the variance is due to differences within participants. This means that there is considerable within-person variation in SOL. This is natural: Sleep varies from night to night in most of us. Only those with a very rigid sleep/wake pattern will have high ICCs, and some of the variation in sleep variables is perhaps not even under the control of the individual – awakenings, for instance, occur spontaneously and depend on a range of different factors. Similarly, one cannot decide to fall asleep at a specific time. Sometimes, SOLs will vary more than on other nights, depending on mental and physical stress, external conditions (noise, light, temperature), and comfort. Therefore, the low ICC, indicating that there seems to be substantial variation between nights slept by the same person (within-person variation) should be considered normal.  Previous research shows that there are considerable within-person variations in sleep variables, see for instance:  Mezick, E. J., Matthews, K. A., Hall, M., Kamarck, T. W., Buysse, D. J., Owens, J. F., & Reis, S. E. (2009). Intra-individual variability in sleep duration and fragmentation: associations with stress. Psychoneuroendocrinology, 34(9), 1346-1354.  Dillon, H. R., Lichstein, K. L., Dautovich, N. D., Taylor, D. J., Riedel, B. W., & Bush, A. J. (2015). Variability in self-reported normal sleep across the adult age span. Journals of Gerontology Series B: Psychological Sciences and Social Sciences, 70(1), 46-56.  We chose not to focus on this in the discussion of the manuscript, as the findings are not novel, but ICCs are reported throughout the “Results” section to inform about this to the readers who find it interesting.  Based on the Mplus output for the analyses that we ran, the average cluster size for the ICC values is 52.571 for the sex differences analyses, 13.533 for the analyses testing menstrual vs. pre-menstrual phase, and  54.333 for the analyses testing follicular vs. luteal phase, and menstrual bleeding days vs. no bleeding days. This information is now added to the “Results” section of the manuscript (lines 279, 313, 328, 343). We now also provide more information about the ICC values in the “Statistical analyses” section (lines 245-249), and also additional clarification on how to interpret the ICC results in the results section of the manuscript (lines 279-282, 313-316, 328-332, 343-246. |
| Figure 1 is not particularly useful and could be supplemental material. | We have now moved Figure 1 to Supplementary material. |
| Figure 2 is very informative showing differences between men and women. It would be helpful to include the women’s data by menstrual cycle phase as part of this figure or as a new figure. | Thank you for your comment. We have now included a new figure (Figure 2, line 354), showing the variations in sleep across the different phases of the menstrual cycle in female athletes. |
| Please include Cronbach alpha for PSQI in your sample and whether it differed by sex. The absence of statistically significant differences may reflect the small N for women (n=15) and perhaps effect sizes should be included. | Cronbach alpha has now been calculated for the entire sample, and broken down by sex. This is provided in the “Materials and Methods / Instruments / Pittsburgh Sleep Quality Index” section, lines 220-221.  In this manuscript, we use R^2^ values to estimate the effect sizes. We use the guidelines from Ferguson (2009) to interpret these, and assign our results practical relevance (lines 254-255) . R^2^ values and their interpretations are now more actively used throughout the discussion. |
| Discussion may require a little revision if results about sleep stage percentages differ from minutes. Discussion mentions levels of discomfort, which raises the issue of medications taken by the women during menses. | The analyses of light, deep and REM sleep in %, and REM latency, uncovered new significant associations. Therefore, we have revised and re-structured the discussion based on the new findings.  Due to the number of new significant associations uncovered in the additional requested analyses, we have slightly amended the way we discuss the results. Unlike the previous version of the manuscript, we now focus the discussion on significant findings only.  Indeed, we agree with the point you are raising about the use of pain medication among menstruating female athletes. We unfortunately did not collect information about this, but we discuss it in relation to the reported findings of athletes’ menstrual symptoms and side effects (e.g., lines 418-423). |
| Discussion of comparisons with the prior research is included with possible explanations for differences, but comparisons focus on Driver #21 findings only. Thus, it is not clear if this study supports other studies that also conflict with Driver’s findings, or if Driver’s results also conflict with others. Driver’s participants were not in their own home, and had more uncomfortable PSG electrodes every-other night in a laboratory, creating the potential for first-night effects, so comparisons of SE would be useful in the discussion; was their overall SE worse or better than women in this sample studied at home with less invasive equipment? It would help readers if discussion compared results with other studies besides Driver’s one study, for example, Baker et al PSG in the lab, Lee et al PSG in the lab (1990) or PSG in the home (2000), or Parry et al PSG young women controls. | Thank you for this comment. Comparisons with previous data are challenging, as methodologies are highly variable and unstandardized. However, we have now done our best to expand the discussion of the findings by referring to papers other than the Driver #21 paper. We hope that the scope of the discussion is now sufficient.  We have now gone through in detail the clinical reviews by Parry and colleagues (2006) and Driver and Baker (1998) to make sure we did not miss out on any publications that investigated variations in sleep throughout the menstrual cycle among healthy, normally menstruating women. |
| The conclusion that this manuscript provides “long-term” data is a bit of a stretch, as that would usually mean over more time than just 2 menstrual cycles, but enough to have, for example, PSQI self-report at 6-month intervals. | We have now removed the words “long-term” from the abstract. In addition, we have edited the abstract in line with the new results, and changed the order in which we present the results related to the menstrual cycle slightly, in order to be consistent with the order in which the results are presented in, in the main body of the paper: 1) bleeding days vs. non-bleeding days, 2) menstrual vs. pre-menstrual phase, and 3) follicular vs. luteal phase. |
